# Information and the Origin of Qualia

**DOI:** 10.3389/fnsys.2017.00022

**Published:** 2017-04-21

**Authors:** Roger Orpwood

**Affiliations:** Centre for Pain Research, Department for Health, University of BathBath, UK

**Keywords:** qualia, consciousness, Shannon information, semantic information, neural networks, attractors

## Abstract

This article argues that qualia are a likely outcome of the processing of information in local cortical networks. It uses an information-based approach and makes a distinction between information structures (the physical embodiment of information in the brain, primarily patterns of action potentials), and information messages (the meaning of those structures to the brain, and the basis of qualia). It develops formal relationships between these two kinds of information, showing how information structures can represent messages, and how information messages can be identified from structures. The article applies this perspective to basic processing in cortical networks or ensembles, showing how networks can transform between the two kinds of information. The article argues that an input pattern of firing is identified by a network as an information message, and that the output pattern of firing generated is a representation of that message. If a network is encouraged to develop an attractor state through attention or other re-entrant processes, then the message identified each time physical information is cycled through the network becomes “representation of the previous message”. Using an example of olfactory perception, it is shown how this piggy-backing of messages on top of previous messages could lead to olfactory qualia. The message identified on each pass of information could evolve from inner identity, to inner form, to inner likeness or image. The outcome is an olfactory quale. It is shown that the same outcome could result from information cycled through a hierarchy of networks in a resonant state. The argument for qualia generation is applied to other sensory modalities, showing how, through a process of brain-wide constraint satisfaction, a particular state of consciousness could develop at any given moment. Evidence for some of the key predictions of the theory is presented, using ECoG data and studies of gamma oscillations and attractors, together with an outline of what further evidence is needed to provide support for the theory.

## Introduction

The really challenging problem in consciousness studies is to find an answer to the question of the origin of subjective experience itself. Of all the large number of articles published on the subject of consciousness most explore the organization of consciousness (e.g., Baars, 1988; Dehaene et al., 1998), or the neural activity that correlates with it (e.g., Rees et al., 2002; Koch et al., 2016). Such work sets the scene for how we are able to have experiences, how the brain can organize itself such that conscious experience results. But the nagging question of how these physical neural activities can give rise to a phenomenal outcome is rarely addressed. Miller has argued (Miller, 2014) that the main emphasis in consciousness science should be on seeking the neural constitution of consciousness, the minimally necessary substrate, but even this (as he admits) would not provide an *explanation* of conscious experience.

There have been some attempts to explore the origin of phenomenal experience. The idea of representational re-description, where a system is able to reflect on its own internal states (Clark and Karmiloff-Smith, 1993), was an early proposal that attempted to map particular kinds of behavior onto the specific properties of phenomenal experience, and led to ideas such as the radical plasticity thesis (Cleeremans, 2008), where a system is able to re-describe its own activities to itself. Indeed the whole concept of meta-cognition, where a system observes its own internal states leading to higher-order representations, also follows this theme of self-reflection (Pasquali et al., 2010). Self-reflection is also at the core of the attention schema theory (Graziano and Kastner, 2011) which proposes that subjective experience is the brain’s internal model of the process of attention, and the authors have presented some evidence to support their ideas (Webb and Graziano, 2015).

However, there is no theoretical account that shows a direct mechanism whereby certain neural activities should lead to a phenomenal outcome. This article is one attempt to link the purely physical with the phenomenal, and it builds on a previous article on the topic (Orpwood, 2013).

The article looks at the generation of qualia. Qualia are often limited to sensory experiences in many author’s definitions. However this article takes a broader view and reflects the definition of qualia preferred by the philosopher Flanagan. He defines the wider sense of qualia as any experience with subjective, first-person, phenomenological feel (Flanagan, 1992). In this definition sensory qualia are just a subset of all the phenomenal experiences that constitute consciousness. There is an underlying assumption in this definition that there is a common cause for all these experiences. The cause of sensory qualia is just the same as all the other experiences, it just happens to be focussed on sensory perception.

The article’s argument is strongly based on an information-processing analysis of cortical network function. Information approaches to understanding consciousness have been pioneered by Tononi (2004) who has argued that conscious states are characterized by the fact that they involve information that is highly integrated and highly differentiated. He developed useful quantifying measures for the informational relationships generated by networks (Balduzzi and Tononi, 2009). However, rather than characterizing consciousness from an information perspective, this article tries to more directly explore the relationships between purely physical information and semantic information that is at the heart of qualia. It aims to show that, in certain circumstances, the processing of information in local cortical networks should lead to qualia.

## Information in The Brain

### Two Kinds of Information

Information is at the core of everything that goes on within neural structures and yet the vast majority of neuroscience studies use a traditional engineering-based definition of what information is. The studies of Shannon over half a century ago (Shannon, 1948) laid the foundations for this understanding. The kind of analysis that is possible using these techniques clearly applies to the information-processing abilities of the brain. Information in Shannon’s analyses is physical information. It is the binary pulses in a communication bus, the pixels on a screen, the written words on a page, etc. The key processes going on in the brain of course involve such physical information. A vast amount of physical information is generated and transferred using local chemical and electrical changes within cells, and electrical and chemical events to communicate this information between cells. The vast bulk of experimental neuroscience is engaged in monitoring these information generators and communicators. But there is another side to the importance of information in a neural context which seems to cause some concern (e.g., Pockett, 2014), and that is the importance of semantic information. Semantic information is the meaning associated with physical information. It is the message that is embodied in, and communicated by, the physical information. In the brain a key aspect of semantic information is the message embodied in the firing of action potentials. It is the meaning conveyed by the massive inter-neuronal communication and processing of physical information. All the information communicated via action potentials throughout the nervous system clearly underlies the inner mental world in some way but the link between these purely physical events and the wealth of inner meanings that they underpin is bewildering. But this problem is at the heart of neuroscience. How does the physical information of neuronal firings relate to the semantic information of the meanings of our inner world?

This understanding is crucial to the understanding of consciousness. Chalmers (1996) in his seminal book proposed the fundamental principle that information has both a physical and a phenomenal aspect. Our conscious inner world is comprised of meanings. It is the way we perceive our environment, and it is the way we experience our inner thoughts. All the qualia that constitute our inner conscious world are aspects of information, but they are aspects of semantic information. They are in some way related to the physical information processing taking place in the firings of our neurons. In order to understand the cause of qualia we need to explore the link between the physical information of neuronal firings and the semantic information of qualia.

This article reflects earlier work (Orpwood, 2013) by defining two new labels for the two kinds of information in the brain that were discussed above. These labels aim to more strongly separate them. First of all the physical information present in the brain is given the label of “information structure”. Information structures are the physical activities of cell firings and trains of action potentials. They are the form through which information is communicated, processed and stored within our brains. Second, the semantic information is given the label “information message”. Information messages are what the physical activity in the brain is all about, what it means to us.

### Transformations between Information Structures and Information Messages

**Figure 1:**
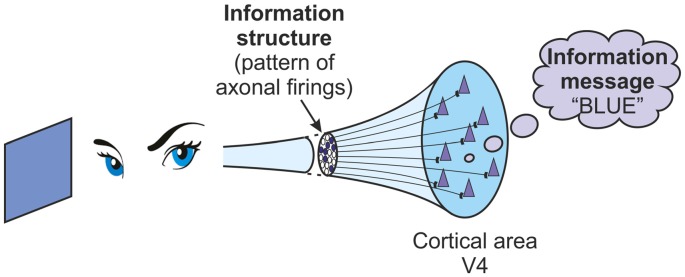
**A brain attending to the color blue receives a pattern of inputs to the V4 area of its visual cortex.** This pattern is an information structure. Its meaning to the brain, “blue”, is an information message.

In order to explore the links between information structures and information messages, it is important to examine the transformations that can occur between them. There are two clear transformations. The first of these is that information structures, in some way, *represent* information messages. Information structures in the brain, in the form of neuronal firings, in some way represent meaning to us. As an example consider firing activity in the V4 area of the visual cortex, the color processing area. If the brain is attending to a blue object then there will be patterns of firing occurring in the V4 area. It is neural activity, and that is just an information structure. But the meaning of the information to that brain, the information message interpreted in some way, is “the color blue” (Figure 1). In some way the information structure of firing activity in networks in V4 represents the information message “the color blue”. As a further example consider the particular information messages we refer to as memories. They are probably embodied in the long-term storage of variable input sensitivities in cortical pyramidal cells. But these variable sensitivities aren’t memories. They can’t be “that wonderful holiday we had last year”. They are just information structures. But they can represent memories. The information structures in place in the variable input sensitivities of cortical pyramidal cells can represent the information messages of memories. Information structures represent information messages. In order to examine the way that qualia are generated, one key task is an analysis of this representational process.

The second relationship between information structures and messages is that messages result from the *recognition and identification* of information structures. This relationship is less easy to envisage because information messages are such nebulous entities. Consider initially a single neuron. It has been shown that they can recognize spatially distributed patterns in their inputs (Mel, 1992; Orpwood, 1994). If an input pattern is received that the neuron has learnt, then it can lead to a substantial soma depolarization. The more significant the recognition the larger the soma depolarization. Eventually the depolarization can reach threshold and the cell will fire. The firing represents the binary decision communicated to the outside world that this particular input pattern is recognized, as opposed to not-recognized. But the recognizing neuron cannot identify the input pattern in any way. Every recognized pattern leads to the same outcome; a firing. An input pattern cannot have any meaning to the neuron other than “seen before”.

The situation is very different for networks of neurons. Consider a network trained on a particular set of input firing patterns. Whenever it is exposed to a firing pattern that is typical of those it has been trained on it will always generate a characteristic output firing pattern. The network is recognizing the input structure because it generates a response when the structure is there, and generates little when the structure isn’t there. But the network is also identifying the input structure because it is generating a typical output response whenever that particular input structure is received. It only generates that characteristic response when it receives an input structure it has been trained on. If it receives an alternative input structure that it has also been trained on then it will generate a different output response. The input has some identity to the network and it is recognized as such, and the output represents that identity. So networks can interpret incoming information structures as messages if they are able to recognize and identify them.

**Figure 2:**
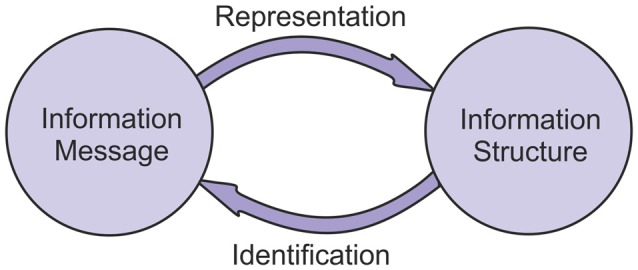
**The reciprocal relationship between information structures and messages.** Information structures represent information messages, and information messages can be identified from information structures.

This analysis shows that the interaction between information structures and information messages is a bi-directional one. Messages can be transformed into structures through a process of representation. Structures can be transformed into messages through a process of identification (Figure 2).

Information structures can be transmitted from an information sender to an information receiver. A sender can generate information structures, and those structures represent something to it. If the sender was responsive, say, to sound, and it had just been exposed to a brief noise, the sender could respond by generating an information structure. That structure would represent the noise to the sender. The sender could then transmit that structure to an information receiver, but it is only the structure that is transmitted. It doesn’t carry the information message with it. The structure that the sender generated could be just a few bits. When those bits are received by the receiver they can mean anything to it. They could mean the noise but they could equally mean the number two, or the shape of a triangle, or even “that wonderful holiday we had last year”. It all depends on how the receiver has been configured. The structure represented the noise to the sender but it could represent any message as far as the receiver is concerned. The communication from an information sender to an information receiver can therefore only be in the form of structures, and not messages.

So some basic conclusions can be drawn from the discussion of information processing so far.

- Information can be in the form of structures or messages.
- The brains physical activity deals with information structures.
- The qualia of our inner conscious world are information messages.
- Structures represent messages.
- Messages can be identified from structures.
- Structures, but not messages, can be transmitted from a sender to a receiver.

## Network Information Transformations

### Basic Network Behavior

A key assumption in this article is that the basic information processing entities in the brain are not individual neurons but ensembles of neurons, or networks as they are labeled in this article. There is a growing conviction that it is ensembles of neurons that are key to the link between neuronal dynamics and their function in information processing, rather than individual cells (Harris, 2005; Buzsáki, 2010; Bharmauria et al., 2016). Some recent evidence for the existence of these ensembles/networks is discussed later. These networks in the cortex consist of a large number of pyramidal cells and their supporting interneurons, which act in a coordinated manner in response to an input barrage. Such networks are able to recognize patterns in their inputs, and can generate their own output patterns of firing activity in response to such recognitions.

Consider initially the behavior of a basic neural network/ensemble in the cortex. The network can demonstrate both the transformations between information structures and messages that were discussed above. At its input, the network is receiving a pattern of action potentials that constitute an information structure. If the input structure is recognized, then the network will react and a number of neurons within it will fire. This firing constitutes the network’s output, and this output is another information structure. This output structure represents the identity of the input to the network. It represents the message, the meaning of the input. That identity depends on the learning that the network has undertaken, or the way that it is configured genetically. Of course the message is not in any way a conscious one. It is just an abstract identity. But if the input structure is identified then an information message is obtained. So a basic network is both an information receiver and an information sender. It is able to transform an incoming structure into a message through a process of identification, and can transform that message into an outgoing structure through a process of representation.

**Figure 3:**
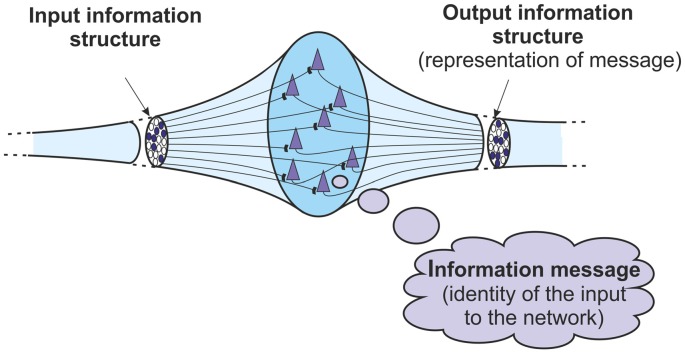
**Basic information processing in a network or ensemble of neurons.** The output information structure represents the information message identified from the input information structure. The information goes from a structure, to a message, to a structure again.

This overall basic mechanism is one that takes place in any cortical network receiving information structures. The input information is a structure. If the network recognizes and identifies that input structure then it will generate an output information structure. That output structure represents the information message, the identity, of the input structure to the network. So within a basic cortical network, it is possible to define more closely the relationship between information structures and information messages. The output structure represents the message obtained when the network identifies its input structure. There is a transformation from structure to message to structure again (Figure 3).

### Communication between Networks

The network can be part of a chain of networks, perhaps in a hierarchy. For each network in the chain the same process as that described above will take place. The network can recognize its input structure and generate an output structure that represents the identity of the input to the network. That structure will be communicated to the next network in the chain. As mentioned above it is only the structure that gets communicated. The next network in the chain can recognize the structure and identify it as a message, depending on the prior learning it has undergone. This process can be repeated along the chain of networks.

The network can also feedback its output to its input again. This local feedback allows the representation that has just been generated to be fed back to the network. It would appear on the surface that if the network recognizes this feedback it would be recognizing its own representation. But it is important to appreciate this situation from the perspective of the network. To an observer the network is receiving its own representation and identifying it. To the network however, although it is receiving its own representation, it of course cannot know this. To the network it is just receiving another input pattern that it can identify or not depending on its prior experience. As was discussed above, the network is both an information sender and an information receiver. If the information sender part generates an information structure and feeds it back to the information receiver part, the only thing communicated is the information structure. There is no message communicated. The information sender cannot inform the information receiver that this structure has the identity “representation”.

The ability to identify representations *as* representations is at the core of the problem of the origin of qualia. After all, the information messages we call qualia are inner representations. A color quale is an inner representation of the hue of light detected. An odor quale is an inner representation of the structure of a volatile molecule detected. How can a representation be identified as a message of “representation” as opposed to just another basic identity? In order to get some insight into how a representation could be identified as the message “representation”, it is necessary to focus on where messages come from. The only statement that can be made is that, to the network, whatever the output represents, that is the message. To the network the message is simply what the output represents. So is it possible for the output to represent a representation? If the output could represent a representation then the message must be “representation”. It is argued below that this is possible when the network settles into an attractor state.

### Attractor States

Consider an individual network that is feeding its output back to its input again. Given this activity the network could achieve an attractor state, most likely a fixed-point attractor. When a network settles into such a stable attractor state its output structure becomes the same as its input structure. The network enters a state of cyclic activity where each output structure generated is the same as the input structure that led to it. In this situation it seems that the network is doing nothing because the output is the same as the input. The transfer function appears to be just unity. This is not the case however. There are complex transformations going on to process the incoming information structure. Each individual neuron is carrying out pattern recognition and interacting with other neurons in the network to conclude with a pattern of firing that happens to be the same as the input.

**Figure 4:**
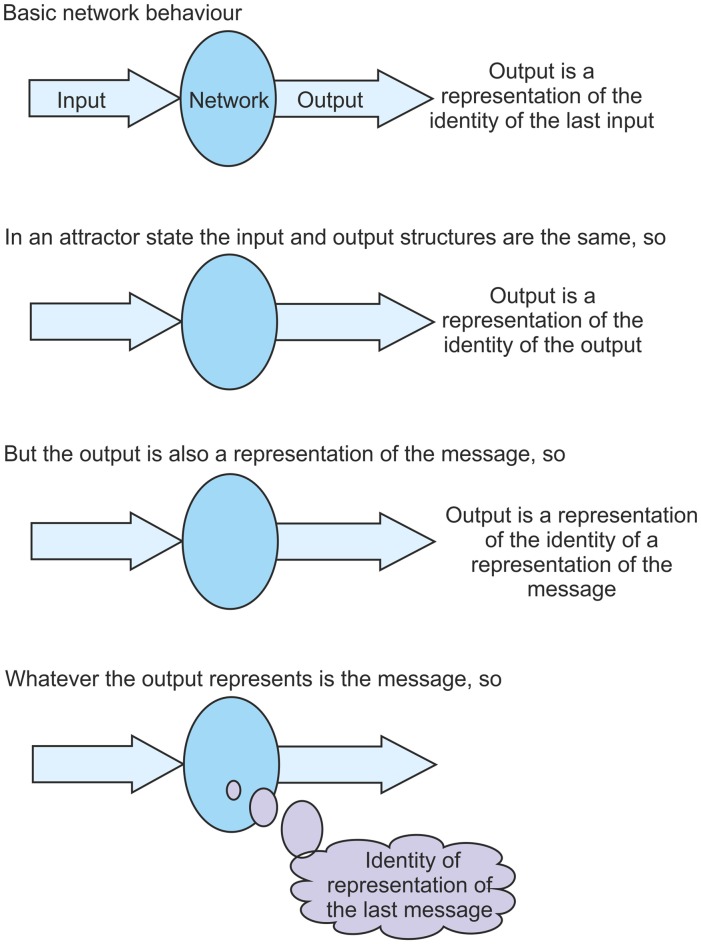
**A key component of the theory presented is that in a settled fixed-point attractor state a network is able to identify its own representations fed back to it** *as*** representations.** This figure aims to clarify the argument for why this is the case. It shows that in an attractor state, as information is cycled through the network, the network is able to identify its fed back input on each pass as a representation of the previous message.

So what is the impact of settling into such an attractor state? As was discussed above, from the perspective of the network the only statement that can be made is that the output from a network is a representation of the identity of the input. But in an attractor state the output is the same as the input. They are the same structures. So in this state the output becomes a representation of the identity of itself. This is quite a difficult concept to appreciate, but it is the case that in this attractor state the output structure is a representation of the identity of that same output structure. The output is a representation of the identity of the output. But, to the network, the output is also a representation of the message. So the output becomes a representation of the identity of a representation of the message. This somewhat complicated statement does tell us something about the message. As was argued above, whatever the output represents, that is the message. So, from the statement, the message must be the identity of a representation of the message. As information is cycled through a network in an attractor state the message obtained each time is the identity of a representation of the last message (see Figure 4). The message is identified as a representation of the previous message. It was argued above that the ability to identify representations *as* representations is at the core of the problem of the origin of qualia. It is shown here that in a stable attractor state this ability is realized. To an observer, whenever information is cycled through a network it is clear that the network is identifying a representation of the previous message. But the network cannot know this until an attractor state has settled. To the network, the message identified each time in an attractor state is “representation of the previous message”.

Consider the start of information being cycled through the network once an attractor state has been achieved. If the network initially receives an input structure and recognizes it then that input structure has some identity to the network, and it recognizes it as “the message”. It generates an output structure that represents the message to the network. If that output structure is fed back the network will identify the feedback as “representation of the previous message”. The original message is “message”, and the second message is “representation of that message”. If the cycling continues then the third message will also be “representation of the previous message”, in other words “representation of the representation of the original message”, and so on. The message is unchanging on each cycle but its nature is evolving. This situation only occurs when the network achieves an attractor status, but it leads to some quite profound properties.

**Figure 5:**
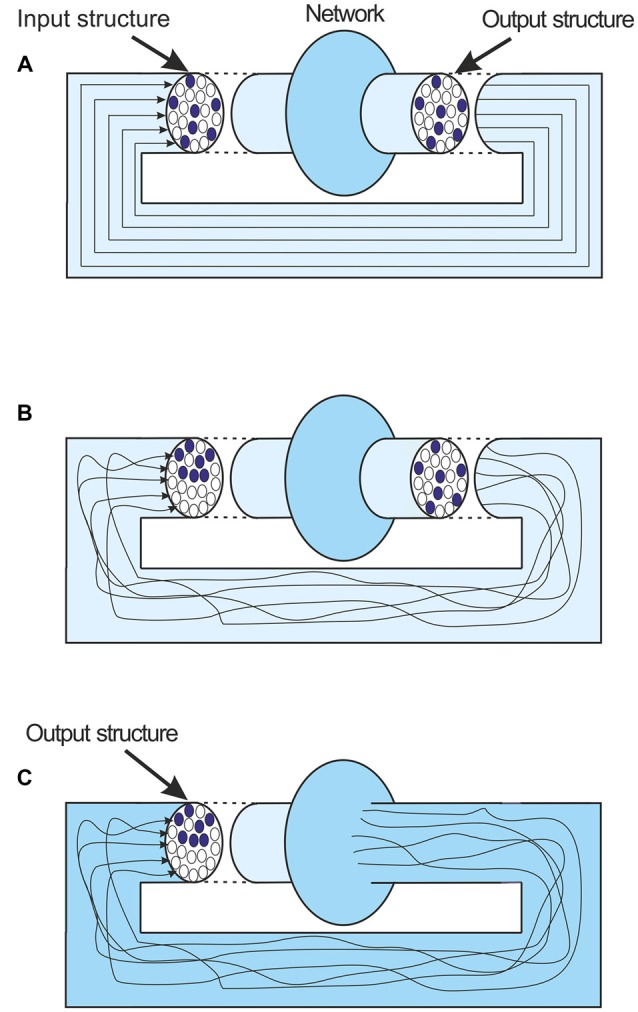
**(A)** An idealized depiction of local feedback in a network. The output structure remains unchanged as it is fed back. **(B)** A more realistic depiction. Feedback axons follow convoluted paths and lead to an input structure that is quite different to the output structure. **(C)** The network boundary is extended to just before the fed back input. The output and the new input are now unchanged. Importantly the output is still a representation of the last message.

There is a complication here that would on the surface seem to dismiss the argument presented. The discussion about attractor states has used very simple illustrations of the nature of the networks involved. In neuro-modeling terms the feedback that leads to attractors, and indeed the mathematical representation of their behavior, is very simplified. The output information structure is considered to be transferred unchanged from the output of the network back to its input again (Figure 5A). But of course in reality this feedback and the connections involved are extremely complicated. The spatial pattern of the feedback activity, the matrix of firing and silent axons, is inevitably going to change as the output activity is fed back to the input (Figure 5B). However the boundary of the network’s activity is not fixed. In simple modeling terms the output is what comes out of the network model, but the boundary of the network model can be extended. If the boundary is extended to just before the input to the network, and the output defined as the structure at that point, then despite the complexities of the feedback connections up to that point, the output structure is fed to the input unchanged (Figure 5C). The important point is that the output at that point is still the network’s representation of the message. Of course it is only when an attractor state has been achieved that the output at that point is the same as the input that led to it.

Without the extra capabilities provided by an attractor state, then a neural recognizer is always starting from scratch with any input it receives. It can only recognize its input as something dependent on its prior learning. With an attractor state this all changes. For each feedback the network will identify its input *as* a representation of its last message. The new message that results from each recognition builds on the previous one and leads to remarkable outcomes.

### Resonant Loops

There is another neuro-anatomical entity whose behavior matches that of attractor states, and which can lead to similar conclusions about the information that is processed by it. This entity is the resonant loop of networks. The idea of resonant loops was pioneered by Grossberg in his Adaptive Resonant Theory (Carpenter and Grossberg, 1987). It was applied to feedforward and feedback interactions between networks in different levels in a hierarchy, such as the sensory processing hierarchies. The activity of networks in one level of the hierarchy is fed forward to higher levels. The higher level makes a judgment about the identity of its fed-forward input, based on prior experiences, and feeds back the outcome of that judgment to the original layer to see if there is any agreement. Depending on the level of agreement the feedback pattern is modified until both the feedforward pattern and the feedback pattern remain unchanged. At that point the higher level judgment of the identity of the input, according to its prior experiences, is correct. Grossberg called this state of stability a resonance. The mechanism involved to adapt the responses of the networks until agreement is achieved was called folded-feedback by Grossberg (Raizada and Grossberg, 2003). Inter-level interactions within a sensory hierarchy are also at the core of predictive coding theories (e.g., Clark, 2013) where adaptions are carried out to reduce an error function reflecting the difference between fed forward activity and fed back judgments as to its identity. These ideas about resonant loops map really well onto the process of perception, and so sensory qualia might well be expected to be linked to these resonant processes.

**Figure 6:**
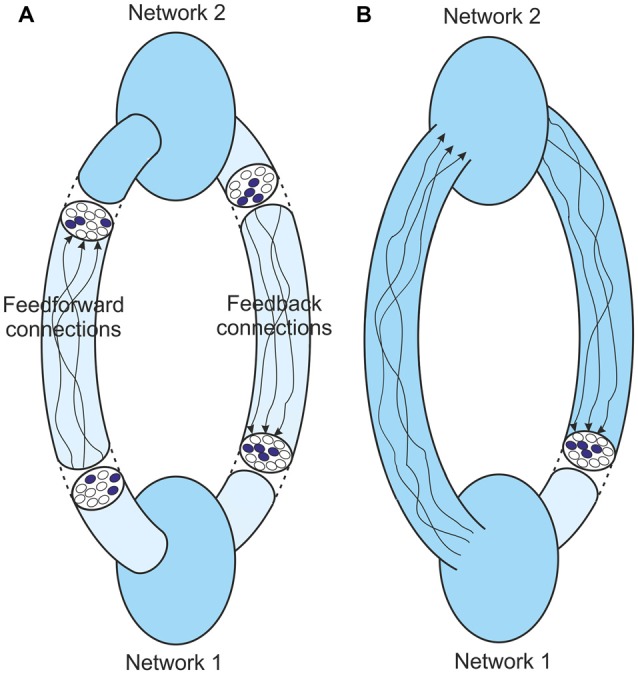
**(A)** Feedback in a two-network loop at resonance. The structures at different points in the system settle to a constant pattern, but the feedforward and feedback paths are convoluted and lead to quite different stable structures at different points. **(B)** The same system with the boundary of Network 1 extended to just before its input. At resonance the input to this network is the same as its output. Importantly the output is still a representation of the last message obtained by Network 1.

Figure 6 shows the core process that goes on in resonant loops. It shows only two interlinked networks, although the likelihood is that perceptual processes are more complicated than this. For the argument to be presented this complexity doesn’t matter. In the simple linked networks the input to Network 1 can be identified by that network, and the output representation of that identity fed forward. This feedforward activity becomes the input to Network 2 which again can identify the input, and the output representation of the identity can be fed back again to Network 1. This iterative activity can continue until agreement is reached and the outputs from the two networks stabilize to unchanging structures (Figure 6A). It is this stable resonant state that underpins the perceptual judgment that is made about the identity of the original input.

This stable resonant state has many parallels with the fixed-point attractor dynamics discussed above. As with the single cortical network the network boundary can be extended to remove the intervening complications between the network’s output and its eventual fed back input (Figure 6B). The eventual feedback to Network 1 is the output from this extended boundary. In the non-stable state whatever input is provided to Network 1 the output from this boundary will be different. In the stable state, whenever Network 1 is provided with this particular input, the same output is generated. So in a stable state this output is a representation of the identity of the input to Network 1.

We can therefore consider Network 1 in isolation. In a stable resonant state it is acting much like an attractor. The output is a representation of the identity of the input. But in the stable state the output is the same as the input that led to it. Therefore the output is a representation of the identity of the output. And that output is a representation of the last message. So the output is a representation of the identity of the representation of the last message. That is what it is to the network. As discussed before, the identity to the network is whatever is represented by the output. So the identity to the network must be the identity of the representation of the last message. In a stable resonant state, as information is cycled through the network, the identity of the input to the network is the identity of its representation of the last message. This result will apply to each network in the resonant loop.

So, to summarize the outcome of information processing in networks, normally a network can only identify its input as a particular “message”. But in two situations involving feedback this changes. The first situation is the achievement of a stable fixed-point attractor state with a single network. The second is the achievement of a stable resonant state involving several interlinked networks. In both these two situations the network identifies its input as “representation of previous message”.

### The Importance of Re-Entrant Activity

Before exploring the generation of qualia it is important to briefly look at how a network can be encouraged to develop an attractor state, or for a hierarchy of networks to achieve a resonant state. Rather than just undergoing one pass of information the networks involved need to be encouraged to repeat this activity via local feedback. A key to this ability is the process of re-entrant feedback, and many authors have explored its impact (e.g., Edelman, 1992; Lamme and Roelfsema, 2000; Bullier, 2001; Pascual-Leone and Walsh, 2001). The underlying principle is that information entering the cortex initially undergoes feedforward transmission through the hierarchy of areas in the sensory cortex and onto prefrontal areas. Concluding network states in higher regions generate information that is then fed back to modulate and integrate the incoming sensory stream. There is growing experimental evidence that the development of conscious percepts is crucially dependent on this re-entrant activity (e.g., Haynes et al., 2005; Silvanto et al., 2005; Boehler et al., 2008). For example Lamme has argued that re-entrant feedback arising from the prefrontal cortex is crucial for visual perception. The prefrontal activity is part of attentional activation following an initial feedforward stream from the visual cortex (Lamme, 2010). Rees has also argued that for conscious vision to take place there is a need for this higher level re-entrant activity (Rees et al., 2002: Rees, 2007). Boly et al. (2011) have also shown top-down projections from frontal cortex are needed for consciousness. However Lamme (2010) has also argued conscious percepts could also arise from re-entrant activity within the visual cortex itself. Supporting this idea of local re-entrant activity Boehler et al. (2008) have shown that early awareness of sensory information can be too rapid to involve prefrontal feedback.

Orpwood (2013) argued that this re-entrant feedback could be crucial to the development of attractor states. Without the facilitation provided by the re-entrant stimulus, network activity, particularly activity involving very local feedback, would quickly fade and attractors could not arise. Only with the stimulus of the re-entrant feedback could local cycling of activity occur long enough to allow attractors to settle. In addition re-entrant feedback has been seen to provide the basis for resonant loops. Lamme and Spekreijse (2000) showed it to be a key element of perceptual organization where higher-level conclusions about the meaning of sensory information are used to fine-tune the lower level building blocks until an agreement is achieved. So for both attractor and resonant states, re-entrant feedback may be needed to instigate, shape and choreograph their development.

## The Generation of Qualia

Orpwood (2013) argued that the ability of networks to identify their inputs as representations of the identity of their previous inputs could lead to the generation of qualia, but it is now felt that the argument presented in that article was overly complicated and not very clear. It also only dealt with the case of stable attractor states. The current article argues that in fact only two cycles of feedback information passage should be needed for qualia to result, and that the process could also involve interlinked networks in a stable resonant state.

**Figure 7:**
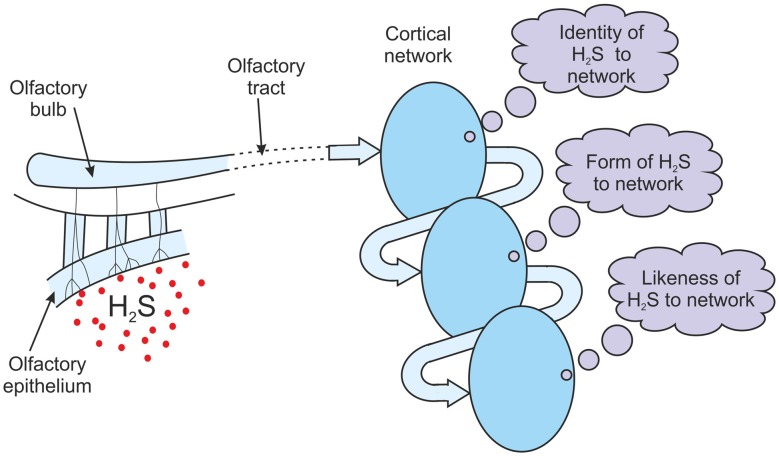
**The generation of olfactory qualia.** Following exposure to H_2_S, information structures from the olfactory tract are cycled through a cortical olfactory network. If attention can lead to an attractor state in this network, the message obtained with each pass of information builds on the previous message. The message evolves from an inner identity of H_2_S, to an inner form of H_2_S, to an inner likeness or image of H_2_S. H_2_S-ness is experienced.

It is perhaps easier to understand how the information processing discussed above could lead to qualia by using an example. Consider the situation of olfactory perception. Assume the nasal epithelium has been exposed to a chemical. The olfactory receptor neurons respond to this exposure, and following local processing of this information in the olfactory bulbs, a pattern of firing is generated along the olfactory tract. This firing pattern is an information structure. There is some debate about whether it is the olfactory cortex or the orbito-frontal cortex that is the site of odor consciousness (Shepherd, 2007), perhaps both, but for the purposes of this argument it doesn’t matter. Assume the information structure being conducted along the olfactory tract reaches the necessary cortical region (Figure 7). If there are ensembles of neurons in this region that recognize the input structure then the recognizing network of cells will generate its own output pattern of firing, an output structure. If there is an output structure generated then that structure must represent the identity of the input to the network. If the chemical that the nasal epithelium was exposed to was hydrogen sulfide then the output structure must be the network’s own representation of H_2_S. It only generates that output structure when the sensors feeding it are exposed to hydrogen sulfide. The output structure represents H_2_S to the network, but not in any conscious sense of course. It is just the network’s own depiction of a chemical that has been detected. It is a structure that embodies the identity “H_2_S” for this network. The incoming input structure was just an abstract set of neuronal firings, but because of the way that the olfactory cortical networks have been configured the output structure is the network’s own representation of the chemical that has been detected. It is a physical embodiment of the identity “H_2_S” for this network (Figure 7).

It is assumed that some attention is being paid to this olfactory input that encourages the development of either an attractor state or a resonant state, as was described above in the section on re-entrant feedback. The output structure will be fed back, either directly or via other networks in a chain or hierarchy. The feedback is a physical structure representing the identity “H_2_S”, but it is identified as a message. It has a specific meaning to the network. What is that message? Before an attractor or resonant state has developed, the message will simply be the identity of a new random input. But, as argued above, once an attractor or resonant state has developed, the message will be “representation of the previous message”. If the previous message was identified as “H_2_S” to the network then the feedback will be identified as its “representation of H_2_S”. The feedback is of course a physical structure, but the identity of that feedback is an inner meaning. The meaning is the network’s own representation of H_2_S, its depiction of the chemical, the inner form of H_2_S to the network. So with this second recognition, the fed back structure is identified by the network as its inner form of H_2_S. Therefore in the case of the olfactory cortex responding to an exposure to hydrogen sulfide, the first message is “identity of H_2_S”, the second message is “inner form of H_2_S” (Figure 7).

As a result of the recognition of the feedback the network will fire again. This second output structure is a representation of the second message, so it is a representation of the network’s inner form of H_2_S. This output structure will be fed back again. The feedback is a physical structure representing the identity “inner form of H_2_S”, but it is identified as a message. It has a specific meaning to the network. What is that message? As an attractor or resonant state has developed the message will be “representation of the previous message”. If the previous message was “inner form of H_2_S” to the network then the feedback will be identified as “representation of inner form of H_2_S”. But a representation of the form of something is a likeness of it, an image of it. The network has communicated to itself an inner likeness or inner image of H_2_S. The feedback is of course a physical structure, but the identity of that feedback is an inner meaning. The meaning is an “inner likeness or image of H_2_S”. To the network it is H_2_S-ness. It is how H_2_S appears to the network, what it is like to it, how it seems to it. The first message was the identity of the chemical, the second message was the form of H_2_S to the network, and the third message is an inner likeness or image of H_2_S (Figure 7). If the brain were able to report the outcome of this process it wouldn’t just be reporting its inner form of H_2_S but how that H_2_S seemed to it. It is identifying an inner experience of H_2_S-ness. It is just an abstract inner sense and not something that can be described to anyone. But from prior learning of words, that inner experience would be given the label of a “smell”. The brain had a quale of the smell of hydrogen sulfide.

If the brain used a different chemical sensing system based on receptors in the tongue rather than the nasal epithelium then a recognized chemical would be interpreted in a similar way. If the tongue sensors responded to glucose in the mouth then the chemical would initially be identified by the cortical networks receiving the information structure from the taste sensors. On first feedback the new input would be identified as the network’s inner form of glucose. On second feedback the new input would be identified as an inner image of glucose. It would lead to an outcome that was how the chemical seemed to the brain. But the inner experience, impossible to put into words, would be different to the olfactory experience. It used different structures in the brain (for a discussion of how qualia can vary depending on the site of generation, see Orpwood, 2010). The outcome would still be how that chemical seemed to the brain, how it experienced it. But in this instance, from learning, the brain would put a label on it of a “taste”. The brain’s experience would just be an abstract inner sense but from knowledge of words it could report the experience as sweetness.

Going back to the example used above of blue color information being communicated to area V4 in the cortex, how will this be interpreted? The initial identity to the network in V4 is “blue”. The network itself cannot of course interpret it as “blue”. For the network it is just an abstract information message. But because of the network’s configuration and learning it recognizes the input information structure. The output structure generated as a result of the initial recognition is the network’s own representation of blue, its inner embodiment of blue. If this is fed back in an attractor state the network would recognize the feedback input as its inner form of the color blue. The network would generate an output that this time was a representation of its inner form of blue. If this second output were fed back and recognized the network would identify it as an inner likeness or image of blue, or blue-ness. It would be how the color blue seemed to the network, how it experienced it. It would be just an abstract, un-describable concept, but from prior learning the brain could give it the label “blue”. A blue quale had been experienced.

Similarly, if the brain received an input to its auditory cortex that resulted from the striking of a tuning fork, how would it respond? The auditory cortex would at some point contain networks that would recognize the tone and generate an output structure that was its inner representation of that particular sound. If that output was fed back in an attractor state it would be recognized as the form of that sound to the network. If the sound was that of middle-C then the second firing would be a representation of the network’s inner form of middle-C. This form representation is then communicated back to the network and identified as an inner likeness. The new message would be how middle-C seemed to the network, how it experienced it. A quale would have been formed that couldn’t be described but it would be middle-C-ness, what middle-C was like to the network. But from learning to express qualia in words it could be given a label of a “sound”. The particular sound of middle-C would be less easy to put into words, but it would have a distinctive quale.

So it is proposed that there could be at any given moment a set of inner experiences related to networks that have settled into an attractor state. Each could lead to an inner experience, a seeming, a quale. There will be qualia associated with activity in the sensory cortices as described. There will also be qualia generated in the amygdala related to emotional experiences, in place cells of the hippocampus related to a sense of place, etc. Only activities in networks that have achieved attractor states would lead to these experiences. As was discussed above and in Orpwood (2013), the development of attractor states could be dependent on re-entrant feedback stimulating networks into this behavior. So attention and other mechanisms would be shepherding the development of attractor states allowing some to develop and inhibiting others. Such control would lead to a kind of brain-wide constraint satisfaction where activity settles down to a set of activities that includes all the networks that are maintained in an attractor state. The set of qualia engendered at that moment could constitute the conscious state of the brain at that moment. So, as underlined in Orpwood (2013), these ideas are quite compatible with more general consciousness theories such as Global Workplace theory. The difference with this article is that it aims to provide a theory about the mechanism of the generation of the phenomenal experience itself.

## Is There Any Evidence for This Behavior?

What would the theory predict would be measurable as qualia are formed? The theory revolves around the activity of local cortical networks and presumes these ensembles are the major functional unit for information processing within the cortex rather than individual cells. It also requires these local networks to undergo cyclic activity as a pre-requisite for qualia to be formed, and requires attractor or resonant states to be formed at the point at which qualia are generated. Is there any evidence for these predictions?

### Networks or Ensembles of Neurons

There is growing evidence that the key information processing entities in the brain are networks of neurons, rather than individual cells. Over 10 years ago Harris argued that the fundamental currency of information processing in the cortex is the spatial pattern of firing activity in assemblies of neurons (Harris, 2005). The idea has been amplified by others (Buzsáki, 2010; Bharmauria et al., 2016). To provide evidence for the role of networks ideally requires studies that examine population activity, and inevitably 2-photon calcium imaging has proven to be a useful tool in this work. Miller et al. (2014) showed specific ensembles of neurons that responded to visual stimuli in awake mice. The same ensemble activity also occurred spontaneously, albeit at much lower levels, as though the ensembles were prior-learnt connected networks. Any given neuron could be part of a number of different ensembles. The stimulus dependence of the activity of pyramidal cell ensembles was also shown by Hofer et al. (2011). They showed that this pyramidal cell activity contrasted markedly with local inhibitory neuron activity which more simply reflected general activity in the locality. Cossell et al. (2015) demonstrated that the majority of the synaptic drive in L2/3 cells is provided by a small number of strong reciprocal inputs between local cells with similar responses to visual inputs. Lee et al. (2016) also showed L2/3 pyramidal cells organized into sub-networks with similar orientation selectivity. As well as these population studies, data from individual units have also indicated the importance of ensembles in information processing. Yoshimura et al. (2005) showed distinct subsets of L2/3 pyramidal cells within a given column that were activated by sensory inputs. Bharmauria et al. (2016) demonstrated specific cell assemblies which discriminated between the orientations of visual inputs, recruiting a different functional network in V1 at all orientations. Such ensembles are exactly the kind of building blocks required to generate qualia according to the theory discussed in this article. Zeki’s original work on micro-consciousness (Moutoussis and Zeki, 1997) showed that, in the visual cortex, movement qualia arise separately to color qualia, implying activity in local networks is at the core of qualia generation. This work has been repeated and investigated further by other authors (e.g., Linares and López-Moliner, 2006; Self, 2014). In an ERP study Bablioni also concluded that synchronized activity in specific cortical networks underlay the generation of qualia (Bablioni et al., 2016).

### Oscillatory Activity

Is there any evidence that these ensembles of neurons are engaged in the kind of cyclic activity required by the theory presented here? Attractor behavior would, by definition, lead to oscillatory activity, and this could be picked up in LFPs or in scalp potentials if sufficient numbers of cells were involved. The involvement of local network feedback would lead to very short feedback paths that would cause quite fast oscillations. This kind of time-course is indicated by various theoretical studies exploring the generation of gamma oscillations in the cortex (e.g., Wang and Buzsaki, 1996). It is highly likely that whenever local attractor oscillations occurred they would be indicated by oscillating field potentials or scalp potentials that were in the gamma frequency range. This does not mean of course that all gamma oscillations are indicative of attractor behavior. Although oscillating cortical potentials such as gamma, or alpha and beta, etc. are often treated as end results in their own right, and therefore a singular phenomenon with a specific function, there is no reason that this should be so. It is more likely that any particular frequency is generated by a number of different activities in the brain where the dynamics of the activity involved happen to lead to that frequency of oscillation (Merker, 2013; Bosman et al., 2014). This point was amplified further by Merker comparing gamma activity to the BOLD signal, as being simply an indicator of local cortical activity, co-varying with cognitive activity rather than being a cognitive operator in its own right (Merker, 2016). The only thing that can be deduced from gamma frequencies is that their fast nature tends to indicate local activity with its short conduction paths. So gamma frequency activity is likely to be generated by attractor behavior, but the occurrence of gamma activity can indicate things going on other than attractors. At the very least however, it would be expected from this theory that activity in the gamma frequency range would be detected in appropriate brain areas if conscious perceptions were reported, and that this activity would be quite local. A technique for distinguishing between the likely gamma activity arising from attractor behavior, and gamma activity arising from other mechanisms, would be extremely useful in testing this hypothesis. Modeling studies have shown that the key current source for the gamma waveforms measured with EEG and MEG arises from activity in L5 neurons, and that activity in L2/3 layers is completely masked when L5 activity occurs at the same time (Lee and Jones, 2013). Given the different feedforward/feedback roles of these laminae in cortical information processing (e.g., Harris and Shepherd, 2015), and their possible impact on consciousness (Orpwood, 2010), the interpretation of gamma activity is not straightforward.

The link between neuronal events and gamma band activity in EEG and MEG recordings is complex (Fernández-Ruiz and Herreras, 2013) but it has for some time been associated with perception, (for reviews see Martinovic and Busch, 2011; Rieder et al., 2011). Early evoked oscillations seem to be linked to feature analysis and later induced activity to perceptual understanding (Tallon-Baudry, 2003; Herrmann et al., 2004). Tallon-Baudry (2003) showed that top-down feedback to sensory areas was associated with increased gamma oscillation during object recognition, and the same activity could be induced by visual imagery. Analysis of evoked and induced gamma responses indicated that both relied on a similar source of neural activity (Porcaro et al., 2011). Visual stimuli have also shown gamma activity to drop to lower levels with sustained stimuli but to continue for the duration of the stimulus (Lowet et al., 2016), also seen in awake monkeys (Swettenham et al., 2009). Reported pain levels seem to reflect the induced gamma band response to pain stimuli (Schulz et al., 2012), with stimuli just above the pain threshold causing increased gamma power compared to those just below (Gross et al., 2007). Visual and acoustic illusions have also led to increased gamma activity in appropriate sensory cortices (Kaiser et al., 2006; Matsuzaki et al., 2012). Gamma synchrony has been highlighted as a key factor in perception, particularly phase synchrony (Fries et al., 2007), and given the proposed importance of ensemble activity in awareness, it is interesting that when functionally connected units were identified in V1 there was an increased gamma power in their responses compared to unconnected neurons (Bharmauria et al., 2015). Attention increases gamma synchronization between visual and other areas (Doesburg et al., 2008), and increases the gamma response to pain (Hauck et al., 2007). However there have been some detractors from the possible link between gamma band activity and awareness. Aru et al. (2012) found that sensory context and prior information led to different gamma responses despite perceptual reports being the same.

Direct recordings from the cortical surface in humans (ECoG) provides a very useful tool for exploring the link between gamma activity and consciousness. There are some useful ECoG reviews (Jacobs and Kahana, 2010; Crone et al., 2011; Lachaux et al., 2012), and Buzsáki et al. (2012) have a good discussion about links between neural activity and ECoG recordings, and show gamma oscillations reflecting firing activity. Using ECoG techniques very local activity in the hippocampus was found to link to conscious recognition (Rey et al., 2014). These authors felt that gamma activity reflected the activity of local cell assemblies as particular concepts were brought into awareness. Also Burke et al. (2014) explored the activity that took place as subjects tried to recall a list of words. They showed three stages; an initial searching stage with high levels of theta, a second recognition stage with high levels of gamma, and a third report stage involving gamma in motor regions. They argued that the theta reflected the searching process whereas gamma reflected the conscious recall. Perceptual face recognition has been used by a number of researchers to look at responses in awake humans. Increases in gamma band activity in facial areas has consistently been found when recognition took place (Lachaux et al., 2005; Fisch et al., 2009). ECoG studies in monkeys also showed large increases in gamma activity over the visual cortex when the animal focussed on viewing an object (Brunet et al., 2015). This activity dropped to low levels following a saccade before returning to high levels again. Gaillard et al. (2009) looked at conscious perception of words. Only conscious stimuli lead to high power gamma, and with gamma synchrony that was very local. The gamma response was sustained throughout the stimulus presentation. Sequence learning has also been explored using ECoG techniques (Madhaven et al., 2015). Gamma power increases occurred during the learning process but as recall improved the gamma power decreased, with new sequences leading to increases again. The gamma power seemed to reflect the conscious content which reduced once a sequence had become learnt. Using their ECoG data, Van Vugt et al. (2014) concluded that sensory percepts are maintained in sensory cortex as synchronized gamma activity which decays but can be re-energized by top down activity from the PFC. Attention has also been linked to these ECoG responses. Lachaux et al. (2008) found gamma band signals decreased in power in one region and increased in another as attention shifted. Ray et al. (2008b) showed both auditory and somato-sensory areas had an increase in gamma power when sensory stimuli were attended to, with an increase over the PFC when this took place.

Some recent articles have underlined the importance of not using a blanket term for higher frequency activity, and have shown that low and high frequency gamma can reflect different responses. Crone et al. (2011) argued that high gamma is more limited in extent and more reliably linked to recognition responses, and also that it was more linked to local synchrony than firing rate changes. The attention-linked increases in gamma power discussed above were also primarily in the high gamma frequencies. Hermes et al. (2015) went further to show that high-level visual perception (faces, buildings) only correlated with high frequency gamma and not lower ones. Lower frequencies could only reliably be elicited by gratings. The high level activity also continued for the duration of the stimulus. Interestingly in macaques it was found with depth electrodes that firing activity led to high gamma rather than low (Ray et al., 2008a). The ECoG literature in general seems to show lower frequency EEG waveforms to be more widespread and related to basic processing. For example Groppe et al. (2013) suggest alpha is related to sensory processing and attention, beta very widespread, and although gamma is prominently observed, it is quite infrequent and localized.

### Attractors

One strand of the theory presented requires that the ensembles of cortical neurons undergo attractor behavior. Although oscillatory waveforms linked to consciousness are a useful indicator, is there more direct evidence that attractors can form in the cortex? There has been an implicit assumption for some 20–30 years that attractor behavior underpins many cognitive functions (Hopfield, 1982; Amit, 1989; Rolls and Treves, 1998). The structure of local cortical networks, and the recurrent collateral connectivity of their pyramidal cells, would imply that they are ideally suited to develop attractor behavior. Many theoretical studies have used this framework. For example Tsodyks (1999) described a theory of hippocampal function that used attractors to define place maps in the hippocampus. In a discussion about motor cortex organization Capaday et al. (2013) concluded that the large amount of recurrent collaterals would enable motor cortex to generate attractors to represent kinetic data. In addition, several theoretical studies of consciousness have been constructed around the generation of attractors (e.g., Mozer, 2009). It was proposed that the contents of consciousness corresponded to transient attractors developing in an interconnected network of computational modules (Mathis and Mozer, 1996). Grossberg claimed that conscious states are a subset of the resonant states that develop between bottom-up and top-down information, and which lie at the heart of his Adaptive Resonance Theory (Grossberg, 1999).

Despite this widespread theoretical thinking, evidence for attractor behavior is only just starting to appear. A key reason for this is that this behavior depends on the activity of whole networks of neurons and evidence has had to wait until the availability of techniques for monitoring large populations of cells. A key technique in these studies had been the use of 2-photon calcium imaging (Wallace and Kerr, 2010). There is evidence that the transient calcium dynamics that are recorded closely reflect cell firing (Kerr and Denk, 2008). However even this technique struggles to follow the detailed time-course of cell firings as it is an indirect measure that can only provide an integrated view of activity over a slower timescale. Miller et al. (2014) demonstrated very clearly in awake mice the repeatedly active ensembles of neurons that arose following visual stimuli, but argued that the time resolution of calcium imaging was not fast enough to determine their detailed dynamics, such as if attractors were developing within the ensembles. And of course these techniques do not lend themselves to work on human subjects, which is key when it comes to exploring conscious experience. Nevertheless evidence for the occurrence of attractor behavior is becoming more widespread. A key aim for future large-scale connectomics projects would be to explore the attractor behavior of cortical networks and the emergent properties that arise (Alivisatos et al., 2012).

Attractor-based theories for hippocampal behavior have been discussed for some time (Rolls, 1996; Tsodyks, 1999) and some evidence has been emerging to support these theories (Poucet and Save, 2005; Knieren and Zhang, 2012). CA3 in particular seems to be able to generate distinct patterns of activity as it is exposed to different environments (Guzowski et al., 2004). One prediction for fixed-point attractors is that as inputs are slowly morphed from one distinct kind to another then the response of the network should not change until about the midpoint of the morphing process when the response should suddenly change from one pattern of activity to another. Wills et al. (2005) showed that as environmental stimuli changed from a round to a square design in stages, there was an abrupt change in the response in CA1 place cells at about the halfway point, as would be expected. Morphed stimuli were also used to explore visual processing in awake monkey IT cortex, and neurons were found that reflected the categorization decision the monkeys made (Akrami et al., 2009). In the auditory cortex of mice, discreet groups of L2/3 cells were found that responded to similar sounds, and as sounds were changed the transitions between the responses of these groups was abrupt, suggesting attractor-like dynamics (Bathellier et al., 2012). The behavior of the awake mice to different sounds could be predicted from the group activities, underscoring their behavioral relevance. In a comment on this work Harris (2012) felt that the firing of the cell assemblies resembled so-called “bump” attractors. However others have shown more progressive changes as environmental stimuli are morphed in this way (Leutgeb et al., 2005). Using a different technique (*in vivo* multi-electrode recordings in pre-motor cortex), Mattia et al. (2013) recorded stereotypical network activity that the authors felt provided compelling evidence that motor plans resulted from attractor activity in local networks as a result of local synaptic reverberation.

So the evidence for the development of attractor states is quite thin on the ground, despite the widespread assumption that it is a common occurence. Measurement techniques at the moment are not quite up to determining whether there are coordinated cycles of firing within ensemble activity. What is really needed is evidence for the generation of attractors during conscious report, and for no conscious experience when attractors do not develop. This can only be done in awake humans but there is no evidence to date with ECoG studies or LFPs for attractor activity linked to conscious report. An indirect signature of attractor behavior using EEG or MEG activity would of course make such studies a lot easier.

### What Would be Needed to Provide Further Evidence?

Despite the overwhelming likelihood that all higher animals experience a degree of consciousness, the only animals we can be a 100% certain about are humans. Therefore it is necessary ultimately to measure activity in humans that underpins conscious experience. For the theory presented here that evidence has to come from monitoring the activity of networks of individual cells, with sub-millisecond resolution, to see how they behave during conscious acts and how that differs to unconscious acts. Such work would necessarily have to remove co-varying activity relating to such things as allocation of attention, activity relating to the reporting process, anticipation, etc. Techniques for population monitoring are of course developing fast, with the pioneering use of 2-photon calcium imaging. At present this technique is not quite fast enough to explore the detail firing activity of cells in networks but this is surely not far off. In the first instance such techniques can be usefully used with higher mammals who are strongly suspected of having conscious experience. Strong pointers would result from monitoring local activity such as that described in this article as the animal indicated a perception as opposed to not indicating a perception. If in parallel with such measurements a signature of that activity could be defined using EEG, MEG or ECoG that would enable human experiments to look for those signatures. Ultimately though it will be necessary to find a technique that can be used in humans, perhaps an ethically acceptable form of light imaging, that can detect the local activity described and to show that it occurs only with conscious awareness.

## Author Contributions

This article presents work carried out by the sole author (RO), and was written by him.

## Conflict of Interest Statement

The author declares that the research was conducted in the absence of any commercial or financial relationships that could be construed as a potential conflict of interest.
